# Spatial examination of social and environmental drivers of Middle East respiratory syndrome coronavirus (MERS-CoV) across Kenya

**DOI:** 10.1007/s10393-024-01684-9

**Published:** 2024-06-25

**Authors:** Ted J. Lawrence, Geoffrey K. Kangogo, Avery Fredman, Sharon L. Deem, Eric M. Fèvre, Ilona Gluecks, James D. Brien, Enbal Shacham

**Affiliations:** 1 Taylor Geospatial Institute, St. Louis, MO USA; 2 College for Public Health and Social Justice, Saint Louis University, St. Louis, MO USA; 3 Washington University, St. Louis, MO USA; 4 Institute for Conservation Medicine, Saint Louis Zoo, St. Louis, MO USA; 5 University of Liverpool, Liverpool, England, UK; 6 International Livestock Research Institute, Nairobi, Kenya; 7 University of Kentucky, Lexington, KY USA

**Keywords:** emerging infectious diseases, climate, land use, spatial analysis

## Abstract

Climate and agricultural land-use change has increased the likelihood of infectious disease emergence and transmissions, but these drivers are often examined separately as combined effects are ignored. Further, seldom are the influence of climate and agricultural land use on emerging infectious diseases examined in a spatially explicit way at regional scales. Our objective in this study was to spatially examine the climate, agriculture, and socio-demographic factors related to agro-pastoralism, and especially the combined effects of these variables that can influence the prevalence of Middle East respiratory syndrome coronavirus (MERS-CoV) in dromedary camels across northern Kenya. Our research questions focused on: (1) How MERS-CoV in dromedary camels has varied across geographic regions of northern Kenya, and (2) what climate, agriculture, and socio-demographic factors of agro-pastoralism were spatially related to the geographic variation of MERS-CoV cases in dromedary camels. To answer our questions, we analyzed the spatial distribution of historical cases based on serological evidence of MERS-CoV at the county level and applied spatial statistical analysis to examine the spatial relationships of the MERS-CoV cases between 2016 and 2018 to climate, agriculture, and socio-demographic factors of agro-pastoralism. Regional differences in MERS-CoV cases were spatially correlated with both social and environmental factors, and particularly ethno-religious camel practices, which highlight the complexity in the distribution of MERS-CoV in dromedary camels across Kenya.

## Introduction

A combination of social and environmental factors often drives the emergence and spread of infectious diseases (Heffernan, 2018). In particular, climate and agricultural land use intersects in ways that influence environmental conditions (Brodie, 2016), which can trigger the emergence and spread of infectious diseases. Specifically, ambient temperature affects infection rates, reproduction, and incubation time of pathogens, with higher temperatures accelerating pathogen maturation (Baker et al., 2022; Semenza et al., 2022). Further, agriculture for food production has been associated with more than 25% of all infectious diseases and more than 50% of all zoonotic infectious diseases that have emerged in humans (Rohr et al., 2019). Lastly, the loss of biodiversity and the ensuing loss of host heterogeneity due in part to climate and agricultural land-use change have been linked to disease susceptibility and transfer (Heffernan, 2018).

In East Africa, changes in climate (i.e., hotter and drier trends) and agricultural land-use change have increased the likelihood of infectious disease emergence and transmission, such as ebola, Usutu, chikungunya, o’nyong-nyong, Rift Valley Fever and crimean-congo hemorrhagic fever viruses (Duygu et al., 2018; Fanelli & Buonavoglia, 2021; Fenollar & Mediannikov, 2018; Muturi et al., 2023; Pandit et al., 2022). Further, a combination of climate, agricultural, and economic changes is supporting the spread of emerging pathogens from East Africa into the Middle East and Europe (Ryan et al., 2019; Xiao et al., 2015). Still, East Africa is considered one of the most at-risk regions in Africa to the impacts of climate change as the livelihoods of a large proportion of the region’s population depend on rain-fed agriculture (Serdeczny et al., 2017). Agro-pastoralists who depend on both livestock keeping and rain-fed crop production are considered the most vulnerable groups to climate change (Hughes & Anderson, 2020). Concurrently, population growth rates in East Africa are among the highest in the world, which increases the pressure for land conversion, and specifically for the expansion of cropland that encroaches on wildlife habitat (Bullock et al., 2021). In all, hotter and drier conditions, population growth, cropland expansion, and encroachment on wildlife habitats can exacerbate infectious disease emergence and transmission (Lee-Cruz et al., 2021).

Despite the combined influence that climate and agricultural land use has on emerging infectious diseases, these drivers are often empirically examined separately and the potential combined effects are often missed (Brodie, 2016). Seldom, the influence of climate and agricultural land use has been examined related to emerging infectious diseases in a spatially explicit way at regional scales. This is especially true for Middle East respiratory syndrome coronavirus (MERS-CoV) that is prevalent in dromedary camels across Kenya where climate and agricultural land-use change is conspicuous (Lawrence et al., 2023a, b).

MERS-CoV is an infectious zoonotic disease that in humans targets the lower respiratory tract and can lead to multi-organ failure, resulting in death. Dromedary camels have been shown to be a natural reservoir of the virus from where spill-over to humans can occur (Adney et al., 2014). The infection is spread when a person comes into close contact with an infected dromedary camel, or possibly when a person consumes contaminated camel products such as milk and meat. Humans can also spread the virus to each other through very-close contact with infected individuals, similar to the current SARS-CoV-2 transmission (Aguanno et al., 2018). The disease was first detected in humans in 2012 in Saudi Arabia (WHO, 2019). MERS-CoV has spread globally with more than 2,000 human infections resulting in nearly 850 identified deaths in 27 countries across North America, Europe, Asia, and Africa as of December 2019. MERS-CoV remains a high-threat pathogen identified by WHO, but no new morbidity and mortality have been reported in the past several years (WHO, 2018), as mild cases of MERS-CoV may be missed by existing surveillance systems and case fatality rates are currently counted only among the laboratory-confirmed cases (Memish et al., 2020). While no human infections have been documented in Kenya previously, the biophysical environment provides an opportunity to predict the conditions in which this may occur. Infection rates among and between camels and humans have been investigated throughout the Middle East (Reeves, 2015), Africa (Gikonyo et al., 2018; Miguel et al., 2017), and Asia (Saqib et al., 2017), which has provided preliminary mapping of infection risk. Since the discovery of MERS-CoV, serological, and molecular evidence has demonstrated that the virus in dromedary camels is genetically similar to the one occurring in humans. Such evidence confirms the hypothesis that dromedary camels are the primary transmission reservoirs, which shed the virus in high numbers and likely serve as reservoirs for human infections (Adney et al., 2014).

Our objective in this study was to spatially examine the climate, agriculture, and socio-demographic factors related to agro-pastoralism, and especially the combined effects of these variables that can influence the prevalence of MERS-CoV in dromedary camels across northern Kenya. Our research questions were: (1) How has MERS-CoV in dromedary camels varied across geographic regions of northern Kenya, and (2) what climate, agriculture, and socio-demographic factors of agro-pastoralism were spatially related to the geographic variation of MERS-CoV cases in dromedary camels? To answer our questions, we analyzed the spatial distribution of historical cases based on serological evidence of MERS-CoV at the county level and applied spatial statistical analysis to examine the spatial relationships of the MERS-CoV cases between 2016 and 2018 to climate, agriculture, and socio-demographic factors of agro-pastoralism. We hypothesized that the number of MERS-CoV cases was elevated in regions comprising hotter and drier conditions and more extensive agro-pastoral practices.

## Study Site, Data Description, and Methods

### Study Site: Kenya

Located in East Africa, Kenya comprises 8 regions and 47 counties that were established through the revised constitution of Kenya in 2010 (“Appendix A”). Kenya’s lands are categorized as predominantly arid or semiarid (located in the Northern Rift Valley, Eastern, Northeastern, and Coastal regions) with only 15% suitable for agricultural production and roughly 80% being rangelands for the population of roughly 50 million humans (Koeva et al., 2020). Despite the relative aridity, Kenya’s arid and semiarid regions support about 25% of Kenya’s human population, 60% of the livestock population that mostly involves pastoralism, and the largest proportion of wildlife (Ngugi & Nyariki, 2005). Further, Kenya is home to Africa’s third largest population of dromedary camels, which play a vital role in food security (Hughes & Anderson, 2020). Smallholder farmers dominate the livestock sector in Kenya with three main livestock production systems: pastoral; dairying; and ranching (Cecchi et al., 2010). Additionally, agricultural and livelihood practices in Kenya are tightly linked to agro-climatic zones (ACZs), which are the delineation of landscapes into regions with relatively homogeneous and contiguous areas based on similar climate characteristics (Boitt et al. 2014; Kogo et al., 2020; Lawrence et al., 2023a, b; Recha, 2019)). Primarily, the ACZs in Kenya represent a temperature gradient from alpine, to temperate, to tropical regions, and a moisture gradient from humid to arid regions (Gikonyo et al., 2018).

### Data Description

The number of MERS-CoV cases in dromedary camels across northern Kenya were compiled from six previous studies published between 2014 and 2020 (“Appendix B”). We define a ‘case’ for the purposes of this study as a camel testing serologically positive to exposure with MERS-CoV by ELISA. Each of the six studies tested for antibodies to MERS-CoV in dromedary camels and reported the results at the county level (Table 1). All of the cases of MERS-CoV in dromedary camels across northern Kenya that were used in this study were from between 1992 and 2018, those cases from 1992 included archived serosamples. It is important to consider the seasonal variation of disease transmission and particularly with respiratory pathogens, such as MERS-CoV. However, Al-Tawfiq and Memish (2019) analyzed all reported MERS-CoV cases from the World Health Organization between January 2013 and December 2017 and did not observe any seasonality in the occurrence of MERS-CoV. Also, we acknowledge that this study relied on previously collected data that differed in methods and specificity of data, which also assumes equal probability of detecting seropositivity. Further, the county-level finding in this study can vary at smaller more local spatial scales. However, we focus on the broader spatial patterns that can be observed in this type of study.

**Table 1:** Number of Samples and Seropositivity of MERS-CoV Cases by Region, County, and Year in Kenya (Corman et al., 2014; Deem et al., 2015; Gardner et al., 2019; Ngere et al., 2020; Ommeh et al., 2018; Sitawa et al., 2020).

Year(s)	Region	County	Samples	Seropositivity (%)
1992	Rift Valley	Laikipia	22	5
1996	Rift Valley	Laikipia	37	5
1998	Eastern	Isiolo	12	17
1998	Rift Valley	Laikipia	50	0
1999	Eastern	Marsabit	41	78
1999	Rift Valley	Laikipia	175	18
1999	Rift Valley	Turkana	50	14
2000	Eastern	Variable	73	53
2000	Rift Valley	Laikipia	56	4
2007	Rift Valley	Baringo	28	0
2008	Northeastern	Mandera/Wajir	162	56
2008	Eastern	Marsabit	21	57
2013	Eastern	Marsabit	7	100
2013	Rift Valley	Laikipia	375	42
2016–2017	Eastern	Isiolo	403	78
2016–2017	Eastern	Marsabit	370	74
2016–2017	Rift Valley	Turkana	417	68
2016–2017	Rift Valley	Laikipia	181	15
2016–2018	Eastern	Marsabit, Isiolo, Samburu	293	80
2016–2018	Rift Valley	Turkana, Baringo, W. Pokot	156	49
2018	Eastern	Marsabit	493	76

Socio-demographic characteristics and agriculture data were from the Kenya Population and Housing Census, the Socio-Economic Atlas of Kenya, 2nd edition (Wiesmann et al., 2016), and Kenyan Statistical Abstracts (KNBS, 2016, 2017, 2018). Climate data were from Weather and Climate-The Global Historical Weather and Climate Data for Kenya (WC-Kenya 2023). The socio-demographic, agriculture, and climate-related data were contemporaneous with the MERS-CoV cases examined between 2016 and 2018. The socio-demographic, agriculture, and climate-related variables tested as independent variables are shown in “Appendix C.” The georeferenced county boundaries of Kenya were from openAFRICA (2015).

### Data Analysis, Model Evaluation and Selection

**Figure 1:**
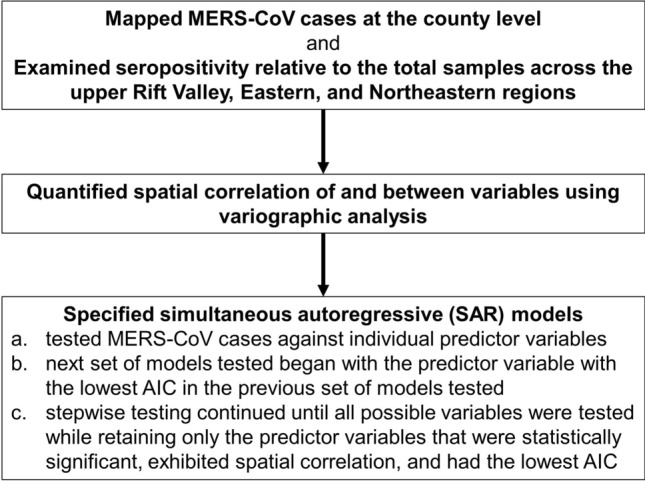
Summary process of spatial modeling and analysis of MERS-CoV and climate, agriculture, and socio-demographic factors related to agro-pastoralism that can influence the prevalence. The initial step involved mapping MERS-CoV cases and examining relative seropositivity across northern parts of regions across Kenya. The next step involved quantifying spatial correlation between the variables. Finally, simultaneous autoregressive models were tested.

We analyzed the data via a combination of non-spatial and spatial analyses using R (R Core Team, 2021), as summarized in Figure 1. Initially, we mapped MERS-CoV cases at the county level in northern Kenya using ArcGIS Pro 2.9.2 (ESRI 2022) and examined seropositivity relative to the total samples across the upper Rift Valley, Eastern, and Northeastern regions during the study period. In the upper Rift Valley region, we included the counties of Turkana, Laikipia, Baringo, and W. Pokot. In the upper Eastern region, we included the counties of Marsabit, Isiolo, and Samburu. In the upper Northeastern region, we included the counties of Mandera and Wajir. We then formally investigated and quantified spatial correlation of and between variables using variographic analysis, which decomposes the spatial variability of observed variables among distance classes. In this process, first, we examined the spatial autocorrelation of MERS-CoV cases. Next, we examined spatial correlation between MERS-CoV cases and (a) socio-demographic variables related to agro-pastoralism, (b) agriculture, and (c) climate variables (“Appendix C”). Given that much of the socio-demographic data were from after 2010 and that the MERS-CoV data in the upper Northeastern region were only from 2008, we focused the spatial correlation analysis of MERS-CoV on 2016 through 2018 in the upper Rift Valley and upper Eastern Regions. The 2016–2018 MERS-CoV data were reported without specifying the date or year that each observation was collected and we therefore spatially analyzed the data as representing a single unit of time (i.e., 3-year time period). Further, we distributed the data evenly between the northern and southern parts of each county to satisfy the practical rule that a variogram should only be applied over a specified distance for which the number of pairs is greater than 30, and because the data were not further spatially identified (Journel and Huijbregts, 1978). We fitted the data using spherical and exponential models and used a distance of between 250 to 350 km, which was deemed conservative, based on the maximum distance of 707 km for the combined upper Rift Valley and Eastern regions of northern Kenya (Journel and Huijbregts, 1978; Crawley, 2012).

After confirming spatial correlation among the MERS-CoV cases and socio-environmental variables, we specified simultaneous autoregressive (SAR) models, a statistical method that augments linear regression models with an additional term to account for the spatial correlation structure in a dataset (Kissling & Carl, 2008). To include the spatial correlation structure of our dataset into the SAR models, we defined neighbors among the northern and southern parts of and between each county based on shared borders and created a spatially weighted matrix. Using shared borders to define neighbors, rather than including counties beyond those with shared borders, allowed us to account for spatial correlation if it diminished over an increasing distance. We weighted each county’s neighbor equally, such that the weights of all neighbors of a sub-county summed to one. Equation 1 shows the general SAR model in matrix form that includes the spatial structure of our dataset.

1 $$ Y \, = \, X{\upbeta } + {\uplambda }W_{u} \, + \, e, $$

where *λ*Wu = the spatial structure (λW) in the spatially dependent error term (*u*). λ = the spatial autoregression coefficient. *W* = the spatial weights matrix. β = a vector representing the slopes associated with the explanatory. variable(s) in the original predictor matrix X. *e* = the (spatially) independent errors.

Our analyses involved testing SAR models in a stepwise process. First, we tested MERS-CoV cases against individual predictor variables. We tended to use variables having absolute values rather than ratios in our modeling and analysis when possible as ratios can result in misleading inferences (Kronmal, 1993). The predictor variables that were statistically significant and spatially correlated with MERS-CoV cases were retained for further testing in the next set of models. The next set of models tested began with the predictor variable with the lowest Akaike information criterion (AIC) value by more than two AIC units in the previous set of models tested. Each of the other statistically significant predictor variables from the previously tested set of models was then individually tested in the new best model at that point in the process. The stepwise testing continued until all possible variables were tested while retaining only the predictor variables that were statistically significant, exhibited spatial correlation, and had the lowest AIC. We evaluated and compared the SAR models relative to each other using the *p* value (with a 10% level of significance) of the likelihood ratio test where a model with no spatial correlation (i.e., λ = 0) is compared to the fitted model with a nonzero spatial correlation parameter (Kissling & Carl, 2008). However, our interpretation of the *p* value was based on a continuum of differing levels of confidence (approximately or greater than 90%) in the null hypothesis (Andrade, 2019). Thus, we interpreted *p* values as a continuous measure of the strength of evidence (Gibson, 2020) with smaller values indicating stronger evidence and large values indicating weaker evidence (Murtaugh, 2014), rather than applying a single, fixed, and arbitrary threshold to assess the probability of false-positive results (Wasserstein and Lazar, 2016). Ultimately, we used the AIC to choose the best performing model.

## Results

**Figure 2:**
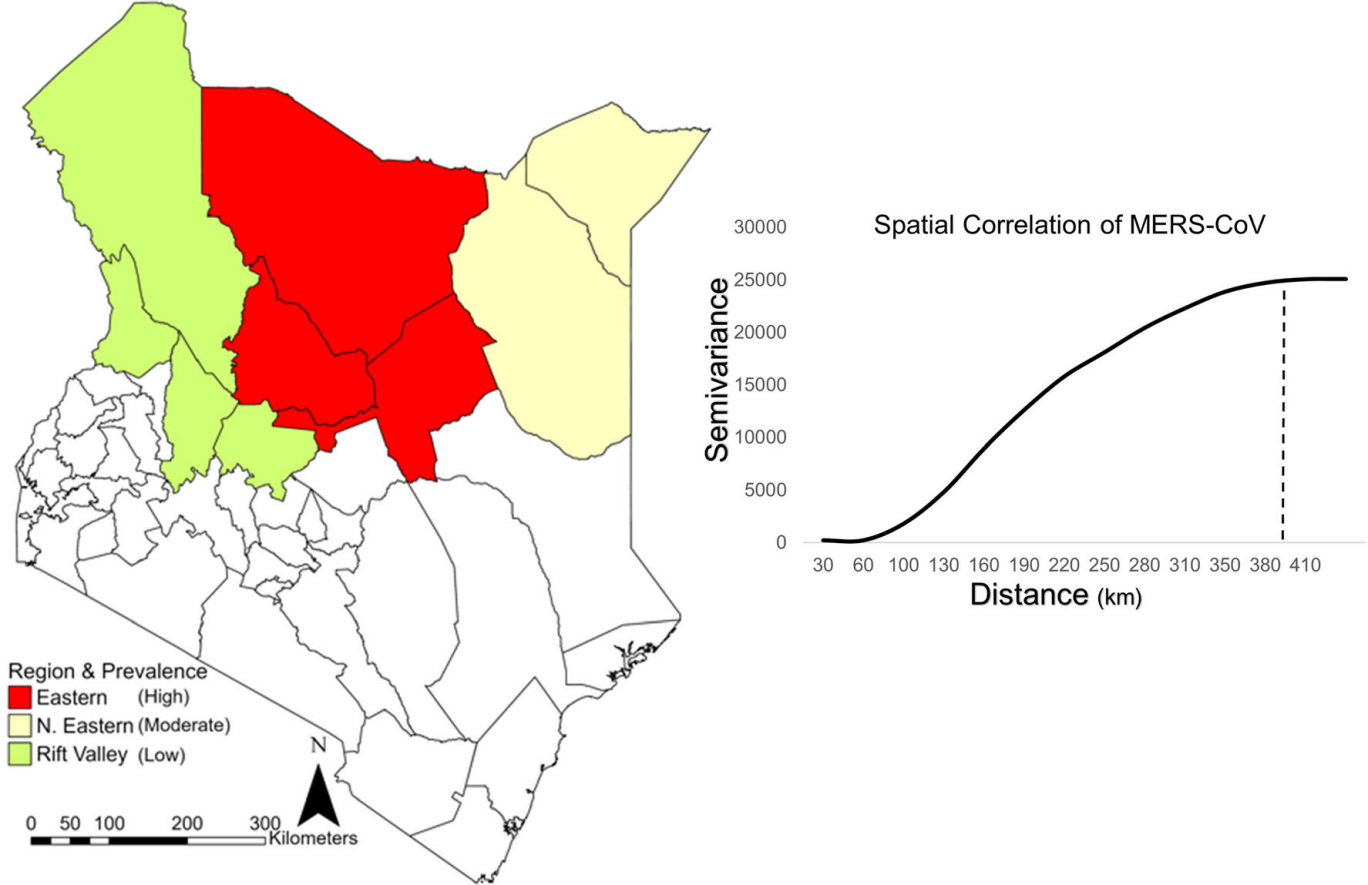
Geographic distribution and spatial correlation of MERS-CoV across northern regions of Kenya. The upper Rift Valley (counties of Turkana, Baringo, W. Pokot, Laikipia) had an average seropositivity of 41% between 1992 and 2018, and an average seropositivity of 51% between 2016 and 2018. The upper Eastern region (counties Marsabit, Isiolo, Samburu) had an average seropositivity of 75% between 1992 and 2018, and an average seropositivity of 77% between 2016 and 2018. The upper Northeastern region (counties of Mandera and Wajir) had a seropositivity of 57% in 2008. The spatial autocorrelation of MERS-CoV extends a distance of approximately 400 km in 2016–2018 across the upper Rift Valley and Eastern regions of northern Kenya. Semivariance is the measure of spatial dependence between two observations as a function of the distance between the observations.

The total number of samples and seropositive cases between 1992 through 2018 in the upper Rift Valley (Turkana, Baringo, W. Pokot, Laikipia) (*n* = 2229, pos. = 914) had a 41% positivity, in the upper Eastern region (Marsabit, Isiolo, Samburu) (*n* = 2486, pos. = 1876) had a 75% positivity, and in the upper North Eastern region (Mandera, Wajir) (*n* = 3506, pos. = 1983) had a 57% positivity (Figure 2). The total number of samples and seropositive cases between 2016 and 2018 in the upper Rift Valley (*n* = 935, pos. = 414) had a 51% positivity, and the upper Eastern region (*n* = 1964, pos. = 1510) had a 77% positivity (Gardner et al., 2019; Ngere et al., 2020; Ommeh et al., 2018; Sitawa et al., 2020). Spatial correlation of MERS-CoV extended a distance of roughly 400 km across the upper Rift Valley and Eastern regions between 2016 and 2018.

The climate, agriculture, and socio-demographic variables (“Appendix C”) that were individually spatially correlated with MERS-CoV cases between 2016 and 2018 and statistically significant according to the p value were included in the SAR models. While all of the seven spatial covariates included in the SAR models did not achieve *p* < 0.10, they were deemed close to the cutoff and important theoretically to assess the spatial relationship (Table 2). According to the AIC, the spatial relationship of ethno-religious camel practices with MERS-CoV cases was significantly better than the other independent variables. Specifically, the *p* value (*p* = 0.015) was below the significance threshold of *p* < 0.05 and the AIC was statistically different (lowest AIC value by more than two AIC units) compared to all the other individually tested independent variables with a − 24.92 change in the AIC from the weakest performing SAR model, which was the spatial relationship of human population with MERS-CoV cases.

**Table 2:** SAR Model Results for Initial Models That Tested Variables in Relation to MERS-CoV in Dromedary Camels Across Upper Rift Valley and Eastern Regions of Kenya.

Model	Variables	Coefficient estimate	Standard error	Autoregressive coefficient (λ)	*P* value	AIC and Δ AIC
	Response					
	Explanatory					
*MERS-CoV prevalence in camels*						
1	(*Intercept)*	119.64	89.97	0.57	0.121	179.5
	Human population	-0.04	0.30			
2	(*Intercept)*	100.19	114.44	0.58	0.079	− 0.01
	Ethnic diversity	2.34	18.76			
3	(*Intercept)*	98.99	82.71	0.59	0.075	− 0.07
	Agropastoral activities	0.43	1.55			
4a	(*Intercept)*	95.59	75.38	0.61	0.062	− 0.37
	Agriculture land density	315.75	502.14			
4b	(*Intercept)*	171.70	85.60	0.59	0.062	− 1.14
	Agriculture land	-0.001	0.001			
5	(*Intercept)*	-506.40	253.12	0.55	0.110	− 5.14
	Avg. air temperature	8.66	3.47			
6	(*Intercept)*	-37.23	45.24	0.74	0.015	− 24.92
	Ethno-religious camel practices	3.33	0.39			

The multi-variate SAR models that were statistically significant and spatially correlated with MERS-CoV cases are shown in Table 3, while all multi-variate SAR models that were tested are shown in “Appendix D” through “Appendix H.” The first combined effect appeared with the inclusion of agricultural land (model 9), which was statistically different from ethno-religious camel practices alone (model 6) based on the AIC, as well as spatially correlated with MERS-CoV cases. When the ethno-religious camel practices variable was combined with agricultural land density rather than agricultural land, model performance declined with a *p* value of 0.019 and an AIC of 156.38 (a + 1.8 change from using ethno-religious camel practices alone). Thus, we focused on testing agricultural land rather than agricultural land density in the models going forward. The SAR model improved, according to the AIC, and a broader combined effect demonstrated when ethnic diversity and agro-pastoral activities were included (model 19). However, the best performing SAR model according to the AIC and the most complex combined effect was when all of the six predictors (model 23) were included and were spatially correlated with MERS-CoV cases and statistically significant. Thus, ethno-religious camel practices and agriculture land were the two initial variables of importance. Next, ethnic diversity and agro-pastoral activities combined with the previous two independent variables were the next set of variables of importance. Finally, average air temperature and human population combined with the previous four independent variables were the final set of variables of importance.

**Table 3:** Results of the Relevant Multi-Variate SAR Models That Tested Variables in Relation to MERS-CoV in Dromedary Camels Across Upper Rift Valley and Eastern Regions of Kenya.

Model	Variables	Coefficient estimate	Standard error	Autoregressive coefficient (λ)	*P* value	AIC
	Response					
	Explanatory					
*MERS-CoV prevalence in camels*						
9	(*Intercept)*	22.51	38.07	0.74	0.029	147.89
	Ethno-religious camel practices	3.31	0.29			
	Agriculture land	− 0.0006	0.0002			
19	(*Intercept)*	− 158.76	45.54	0.74	0.046	138.37
	Ethno-religious camel practices	4.38	0.29			
	Agriculture land	0.0007	0.0003			
	Ethnic diversity	21.74	4.95			
	Agropastoral activities	− 3.47	0.80			
23	(*Intercept)*	− 501.65	65.35	− 1.39	0.008	95.64
	Ethno-religious camel practices	3.09	0.16			
	Agriculture land	0.0001	0.00005			
	Ethnic diversity	12.08	0.82			
	Agropastoral	− 2.22	0.613			
	Average temperature	7.57	1.10			
	Human population	− 0.34	0.08			

## Discussion

This study was conducted to collate data from disparate studies and datasets to provide greater insight to the prediction of MERS-CoV infections that may be facing Kenya, and regions with similar geographies across the world. Most often, the occurrence of infectious diseases has been studied independently, overlooking the potential combined effects of climate change, agricultural land use, and human–environment interaction. Here, we comprehensively and spatially evaluated the influence of various components that may contribute to the emergence of MERS-CoV throughout the northern regions of Kenya. This study highlighted the benefit of bringing together diverse areas of data and research to spatially inform infectious disease risk.

The geographical variations of serological evidence of MERS-CoV in dromedary camels and spatial correlations with the social and environmental variables may be partly attributed to the dominant ethnic groups in the different regions and their particular agro-pastoral management practices. Our results showed low MERS-CoV seropositivity in the Rift Valley region where tribal communities practice a more diversified form of livestock management with camels reared alongside other livestock, such as cattle, sheep, and goats (Iiyama et al., 2008), which could create a dilution effect where the diversity of an ecological community reduces the transmission of a pathogen (Keesing and Ostfeld, 2021). Particularly, the Turkana communities and the Pokot have adapted livestock diversification as part of a long-term adaptation strategy to manage drought and diseases (Opiyo et al., 2015), and tend to acquire cattle and camels through cultural practices, such as dowry rather than directly from markets (De Vries et al., 2006). Thus, tribal communities, such as Pokot, Maasai, or Turkana, have a limited dependence on camels for their economic activity that can result in lower seropositivity of MER-CoV compared to other regions (Deem et al., 2015).

Tribal communities, such as the Somali, Gabra, and Borana, in the eastern and northeastern regions of Kenya primarily focus on camels as a pastoral livelihood strategy due to the camel’s adaptability to arid environments and ability to withstand droughts (Ngere et al., 2020). Also, the Somali primarily inhabit the Northeastern region with high regard for camels for their annual religious practices and ritual migrations (Watson et al., 2016), and a greater number of cultural practices and traditions associated with livestock rearing, trade, and consumption in tribal communities (Dan et al., 2021; Kagunyu et al., 2018). Further, the tribal communities in both the Eastern and Northeastern regions tend to migrate to different areas in search of fresh water and pastures, and restock their camels through direct markets, which can lead to higher prevalence rates of MERS-CoV (De Vries et al., 2006; Hughes & Anderson, 2020).

Recent findings have shown that the expanding arid regions of Kenya have reduced agricultural land use and increased the reliance on pastoralism, and particularly camel rearing (Lawrence et al., 2023a). Also, the increasing trend in air temperatures has resulted in decreased rain-fed agricultural production, and further leading to increased pastoralism (Lawrence et al., 2023b). As a consequence, these combined effects of climate and land-use change can expand the range of locations suitable to a particular pathogen or vector (Baker et al., 2022) and may continue to pose a major challenge in creating favorable conditions for the emergence and transmission of viral zoonotic diseases resulting in the observed high seropositivity rates of MERS-CoV. Similar results of spatial clustering of zoonotic diseases have been studied in different agro-ecological climate zones and socio-demographics of Kenya to identify potential disease hot spots of anthrax (Nderitu et al., 2021). More broadly, Carlson et al. (2022) established a macroecological link between climate change and cross-species viral transmission. Thus, in recent decades, the interconnectedness between global change, such as climate and land-use change—and infectious disease emergence and spread, including MERS-CoV has become a more widely accepted phenomenon (Nova et al., 2022). Overall, climate change across Africa can affect pathogens directly through influencing the survival, reproduction, and life cycle of pathogens, or indirectly by controlling the habitat, environment, or competitors of pathogens and by altering the contact patterns of human-pathogen and human-vector (Chala and Hamde, 2021). These findings are significant as shifting climatic zones continue to impact agricultural systems, while zoonotic diseases like MERS-CoV pose a major risk to communities living in arid regions of Kenya warranting immediate interventions and continued surveillance.

Due to these climatic and agricultural shifts, there is an urgent need to predict where and under what conditions would viral transmission of MERS-CoV occur. These findings suggest that continued monitoring of camels, humans, and their spatial patterns will be an integral component of informing prediction models. We also recommend that more attention should be accorded to the notion of critical environmental thresholds, as opposed to infection thresholds or related warning signals (Baker et al., 2022). Future prediction modeling should therefore aim to establish a macroecological link between climate and land-use change and emerging infectious diseases and transmission (Carlson et al., 2022). Specifically, the application of ecological niche modeling to predict hot spots for emerging infectious disease that include factors such as climate and land-use change can improve location-based risk management (Lawrence et al., 2023c). Further, the application of such modeling approaches to the intersection of climate and agricultural land-use change can be more readily accomplished through the examination of land tenure types that act as a mechanism for shifting agricultural land use and impact geographic patterns of emerging infectious diseases (Buck et al., 2019; Lawrence et al., 2020). These should be factors to consider in epidemiological dynamics studies in other locations worldwide, such as the Amazon in South America and tropical forests in Asia.

This study relied on previously collected data that differed in methods and specificity of data, which also assumes equal probability of detecting seropositivity. Further, the county-level finding in this study can vary at smaller more local spatial scales. Thus, results are limited in power and yet, the broader spatial patterns observed and insights that this type of study can provide are important in the planning and implementation of prediction modeling, as well as location-based risk management.

## Conclusion

Climate and agricultural land-use change have increased the likelihood of infectious disease emergence and transmissions, but these drivers are often examined separately as combined effects are ignored. Further, seldom are the influence of climate and agricultural land use on emerging infectious diseases examined in a spatially explicit way at regional scales. Our objective in this study was to spatially examine the climate, agriculture, and socio-demographic factors related to agro-pastoralism that can influence the prevalence of MERS-CoV in dromedary camels across northern Kenya. We showed that regional differences in MERS-CoV cases were spatially correlated with both social and environmental factors related to agro-pastoral practices, and particularly ethno-religious camel practices, which highlight the complexity in the distribution of MERS-CoV in dromedary camels across Kenya. Overall, this study can provide important insights in the planning and implementation of prediction modeling, as well as location-based risk management.

## Figures and Tables

**Figure 3:**
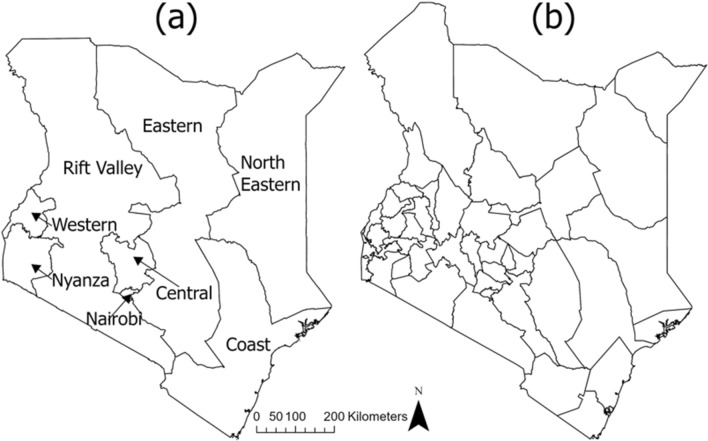
Kenya regional and county maps. Kenya comprises 8 regions (**a**) and 47 counties (**b**).

## Appendix A

See Figure 3.

## Appendix B

Previous MERS-CoV studies in Kenya that report seropositivity.

1. Corman VM, Jores J, Meyer B, Younan M, Liljander A, Said MY, Gluecks I, Lattwein E, Bosch B-J, Drexler JF, Bornstein S, Drosten C, Müller MA (2014) Antibodies against MERS Coronavirus in Dromedary Camels, Kenya, 1992–2013 *Emerging Infectious Diseases* 20(8). 10.3201/eid2008.140596
2. Deem SL, Fèvre EM, Kinnaird M, Browne AS, Muloi D, Godeke GJ, Koopmans M, Reusken CB (2015) Serological evidence of MERS-CoV antibodies in dromedary camels (*Camelus dromedarius*) in Laikipia county, Kenya *PLOS ONE* 10(10):e0140125. 10.1371/journal.pone.0140125
3. Gardner EG, Kiambi S, Sitawa R, Kelton D, Kimutai J, Poljak Z, Tadesse Z, Von Dobschuetz S, Wiersma L, Greer AL (2019) Force of infection of Middle East respiratory syndrome in dromedary camels in Kenya *Emerging Infectious Diseases* 147. 10.1017/s0950268819001663
4. Ngere I, Munyua P, Harcourt J, Hunsperger E, Thornburg N, Muturi M, Osoro E, Gachohi J, Bodha B, Okotu B, Oyugi J, Jaoko W, Mwatondo A, Njenga K, Widdowson MA (2020) High MERS-CoV seropositivity associated with camel herd profile, husbandry practices and household socio-demographic characteristics in Northern Kenya *Emerging Infectious Diseases* 148. 10.1017/s0950268820002939
5. Ommeh S, Zhang W, Zohaib A, Chen J, Zhang H, Hu B, Ge XY, Yang XL, Masika M, Obanda V, Luo Y, Li S, Waruhiu C, Li B, Zhu Y, Ouma D, Odendo V, Wang L-F, Anderson DE, Shi Z-L (2018) Genetic evidence of middle east respiratory syndrome coronavirus (MERS-Cov) and widespread seroprevalence among camels in Kenya *Virologica Sinica* 33(6):484–492. 10.1007/s12250-018-0076-4
6. Sitawa R, Folorunso F, Obonyo M, Apamaku M, Kiambi S, Gikonyo S, Kiptiness J, Njagi O, Githinji J, Ngoci J, VonDobschuetz S, Morzaria S, Ihab E, Gardner E, Wiersma L, Makonnen Y (2020) Risk factors for serological evidence of MERS-CoV in camels, Kenya, 2016–2017 *Preventive Veterinary Medicine* 185:105–197. 10.1016/j.prevetmed.2020.105197

## Appendix C

See Table 4.

**Table 4:** Independent Variables Tested in Spatial Statistical Models to Predict MERS-CoV in Kenya.

Category		
Variable name	Definition	Units
*Socio-demographic*		
Human population	Number of people in each county	People
Human population density	Number of people relative to the area of the county	People/km
Poverty incidence	Number of poor people relative to the total population at the same location	People/people
Poverty severity	Captures how deep poverty is and how difficult it is to climb out of it	*
Religious camel practices	Number of people in a county that include camels in religious practices, such as dowry or ceremonies relative to total county population	People/people
Ethnic diversity	Number of ethnic groups represented in a county	Groups
*Agriculture*		
Agropastoral activity	Number of people in small-scale agriculture and pastoralism	People
Households owning livestock	Number of households owning livestock in a county	Households
Agriculture land	Amount of agriculture land in a county	Hectares
Agriculture land density	Amount of agriculture land relative to the area of the county	Hectares/hectares
Camel population	Total number of camels in a county	Camels
Camel density	Camel per area in a county	Camels/km
Livestock	The number of stock in a county	Livestock
Camel-to-cattle ratio	Number of camels relative to the number of cattle in a county	Camels/cattle
Camel-to-livestock ratio	Number of camels relative to the number of livestock in a county	Camels/livestock
Camel per population	Number of camel per human population in a county	Camels/people
Livestock production	Number of livestock utilized to provide labor and produce various products for consumption	Livestock
Agro-climatic zone 1–3	Humid area that exhibit specific rainfall, temperature, and soil characteristics	km
% of Agro-climatic zone 1–3	Agro-climatic zone 1–3 area relative to the total area of the county	km/km
Agro-climatic zone 4–7	Arid area that exhibit specific rainfall, temperature, and soil characteristics	km
% of Agro-climatic zone 4–7	Agro-climatic zone 4–7 area relative to the total area of the county	km/km
Average temperature	Average monthly temperature in a county	Degrees celsius
Average precipitation	Average monthly precipitation in a county	mm

*For further description and units, see Wiesmann et al., 2016.

## Appendix D

See Table 5.

**Table 5:** Second Set of Models That Tested Variables in Relation to MERS-CoV in Dromedary Camels Across Upper Rift Valley and Eastern Regions of Kenya.

Model	Variables		Coefficient estimate	Standard error	Autoregressive coefficient (λ)	*P* value	AIC and Δ AIC
	Response						
	Explanatory						
*MERS-CoV prevalence in camels*							
6		(*Intercept)*	-37.23	45.24	0.74	0.015	154.58
		Ethno-religious camel practices	3.33	0.39			
7		(*Intercept)*	194.17	146.34	0.74	0.019	− 0.49
		Ethno-religious camel practices	3.95	0.52			
		Avg. air temperature	− 3.63	2.20			
8		(*Intercept)*	− 106.39	54.54	0.74	0.018	− 1.30
		Ethno-religious camel practices	3.43	0.35			
		Ethnic diversity	12.93	6.70			
9		(*Intercept)*	22.51	38.07	0.74	0.029	− 6.69
		Ethno-religious camel practices	3.31	0.29			
		Agriculture land	− 0.0006	0.0001			
10		(*Intercept)*	− 6.99	24.64	0.56	0.240	− 7.77
		Ethno-religious camel practices	3.87	0.31			
		Agropastoral	− 1.91	0.49			
11		(*Intercept)*	25.60	14.53	− 0.27	0.634	− 15.47
		Ethno-religious camel practices	4.09	0.21			
		Human population	− 0.48	0.05			

## Appendix E

See Table 6.

**Table 6:** Third Set of Models That Tested Variables in Relation to MERS-CoV in Dromedary Camels Across Upper Rift Valley and Eastern Regions of Kenya.

Model	Variables	Coefficient estimate	Standard error	Autoregressive coefficient (λ)	*P* value	AIC and Δ AIC
	Response					
	Explanatory					
*MERS-CoV prevalence in camels*						
9	(*Intercept)*	22.51	38.07	0.74	0.029	147.89
	Ethno-religious camel practices	3.31	0.29			
	Agriculture land	− 0.0006	0.0002			
12	(Intercept)	− 217.65	164.42	0.72	0.046	− 0.05
	Ethno-religious camel practices	2.57	0.57			
	Agriculture land	− 0.0009	0.00027			
	Average temperature	4.30	2.89			
13	(*Intercept)*	− 22.48	53.66	0.79	0.013	0.40
	Ethno-religious camel practices	3.39	0.27			
	Agriculture land	− 0.0005	0.00016			
	Ethnic diversity	7.21	5.44			
14	(Intercept)	5.99	31.77	0.64	0.18	0.46
	Ethno-religious camel practices	3.68	0.36			
	Agriculture land	− 0.0002	0.0003			
	Agropastoral	− 1.260	0.923			
15	(*Intercept)*	25.33	14.54	− 0.27	0.618	− 6.83
	Ethno-religious camel practices	4.09	0.21			
	Agriculture land	0.00004	0.00017			
	Human population	− 0.50	0.09			

## Appendix F

See Table 7.

**Table 7:** Fourth Set of Models That Tested Variables in Relation to MERS-CoV in Dromedary Camels Across Upper Rift Valley and Eastern Regions of Kenya.

Model	Variables	Coefficient estimate		Standard error	Autoregressive coefficient (λ)	*P* value	AIC and Δ AIC
	Response						
	Explanatory						
*MERS-CoV prevalence in camels*							
12	(Intercept)	− 217.65	164.42		0.72	0.046	147.84
	Ethno-religious camel practices	2.58	0.57				
	Agriculture land	− 0.0009	0.0003				
	Average temperature	4.30	2.89				
16	(Intercept)	− 171.47	187.71		0.75	0.051	1.83
	Ethno-religious camel practices	2.79	0.75				
	Agriculture land	− 0.0008	0.0004				
	Average temperature	3.13	3.82				
	Ethnic diversity	3.14	7.29				
17	(Intercept)	− 784.30	83.87		− 0.52	0.318	− 20.99
	Ethno-religious camel practices	1.93	0.31				
	Agriculture land	− 0.0005	0.00012				
	Average temperature	13.78	1.50				
	Agropastoral	− 4.13	0.39				
18	(Intercept)	− 407.43	77.45		− 0.27	0.599	− 21.30
	Ethno-religious camel practices	2.54	0.30				
	Agriculture land	− 0.00044	0.00013				
	Average temperature	7.80	1.39				
	Human population	− 0.52	0.05				

## Appendix G

See Table 8.

**Table 8:** Fifth Set of Models That Tested Variables in Relation to MERS-CoV in Dromedary Camels Across Upper Rift Valley and Eastern Regions of Kenya.

Model	Variables	Coefficient estimate	Standard error	Autoregressive coefficient (λ)	*P* value	AIC and Δ AIC
	Response					
	Explanatory					
*MERS-CoV prevalence in camels*						
13	(*Intercept)*	− 22.48	53.66	0.79	0.013	148.29
	Ethno-religious camel practices	3.39	0.27			
	Agriculture land	− 0.0005	0.00016			
	Ethnic diversity	7.21	5.44			
19	(*Intercept)*	− 158.76	45.54	0.74	0.046	− 9.92
	Ethno-religious camel practices	4.38	0.29			
	Agriculture land	0.0007	0.0003			
	Ethnic diversity	21.74	4.95			
	Agropastoral	− 3.47	0.80			
20	(*Intercept)*	− 97.90	16.57	− 0.54	0.384	− 28.77
	Ethno-religious camel practices	4.41	0.09			
	Agriculture land	0.001	0.0001			
	Ethnic diversity	17.95	2.27			
	Human population	− 0.662	0.042			

## Appendix H

See Table 9.

**Table 9:** Sixth Set of Models that Tested Variables in Relation to MERS-CoV in Dromedary Camels Across Upper Rift Valley and Eastern Regions of Kenya.

Model	Variables	Coefficient estimate	Standard error	Autoregressive coefficient (λ)	*P* value	AIC and Δ AIC
	Response					
	Explanatory					
*MERS-CoV prevalence in camels*						
19	(*Intercept)*	− 158.76	45.54	0.74	0.046	138.37
	Ethno-religious camel practices	4.38	0.29			
	Agriculture land	0.0007	0.0003			
	Ethnic diversity	21.74	4.95			
	Agropastoral	− 3.47	0.80			
21	(*Intercept)*	− 53.39	16.58	− 1.09	0.067	− 24.61
	Ethno-religious camel practices	4.10	0.11			
	Agriculture land	0.0003	0.0001			
	Ethnic diversity	14.03	1.81			
	Agropastoral	1.77	0.50			
	Human population	− 0.85	0.06			
22	(*Intercept)*	− 774.68	32.04	− 1.14	0.063	− 32.72
	Ethno-religious camel practices	2.57	0.15			
	Agriculture land	0.00002	0.0001			
	Ethnic diversity	11.50	1.38			
	Agropastoral	− 4.93	0.16			
	Average temperature	12.04	0.62			
23	(*Intercept)*	− 501.65	65.35	− 1.39	0.008	− 42.73
	Ethno-religious camel practices	3.09	0.16			
	Agriculture land	0.0001	0.00005			
	Ethnic diversity	12.08	0.82			
	Agropastoral	− 2.22	0.613			
	Average temperature	7.57	1.10			
	Human population	− 0.34	0.08			
